# Evaluation of the need for routine clinical testing of *PALB2* c.1592delT mutation in BRCA negative Northern Finnish breast cancer families

**DOI:** 10.1186/1471-2350-14-82

**Published:** 2013-08-13

**Authors:** Maria Haanpää, Katri Pylkäs, Jukka S Moilanen, Robert Winqvist

**Affiliations:** 1 Laboratory of Cancer Genetics and Tumor Biology, Department of Clinical Chemistry and Biocenter Oulu, Institute of Diagnostics, University of Oulu, P.O. Box 5000, 90014 Oulu, Finland; 2 Laboratory of Cancer Genetics and Tumor Biology, Northern Finland Laboratory Centre NordLab, Oulu University Hospital, P.O. Box 500, 90029 OYS, Oulu, Finland; 3 Department of Clinical Genetics, Institute of Clinical Medicine, University of Oulu, P.O. Box 5000, 90014 Oulu, Finland; 4 Department of Clinical Genetics, Oulu University Hospital, P.O. Box 23, 90029 OYS, Oulu, Finland

**Keywords:** Hereditary breast cancer predisposition, *PALB2*, Genetic counseling

## Abstract

**Background:**

Testing for mutations in the *BRCA1* and *BRCA2* genes among high-risk breast cancer patients has become a routine practice among clinical geneticists. Unfortunately, however, the genetic background of a majority of the cases coming to the clinics remains currently unexplained, making genetic counseling rather challenging. In recent years it has become evident world-wide that also women carrying a heterozygous germline mutation in *PALB2* are at significantly increased risk of getting breast cancer. We have previously studied the clinical as well as biological impact of the *PALB2* c.1592delT founder mutation occurring in about 1% of Finnish breast cancer patients unselected for their family history of disease, and our results demonstrated a 40% increased breast cancer risk by age 70 for female mutation carriers. Thus, this relatively common mutation in *PALB2* is associated with a high risk of developing breast cancer. The aim of the current study was to analyze whether female index individuals of breast cancer families who had tested negative for germline mutations in *BRCA1*/*BRCA2* as part of genetic counseling services should be offered mutation testing for *PALB2* c.1592delT.

**Methods:**

The study cohort consisted of altogether 223 individuals who had contacted the Department of Clinical Genetics at the Oulu University Hospital in Finland between the years 1997 and 2011 for counseling on hereditary breast and/or ovarian cancer risk. 101 of them met our inclusion criteria. Of these, 10 persons were now deceased, but 6 of them had participated in one of our previous studies on *PALB2*. Seventy (77%) of the remaining 91 persons responded positively to our study invitation. Chart review of updated pedigree data led to the exclusion of 14 further individuals not meeting the selection criteria.

**Result:**

Of the 56 alive affected female individuals screened for *PALB2* c.1592delT, altogether two (3.6%) tested positive for this mutation. In addition, of the previously tested but now deceased 6 persons eligible for the current study, one more mutation carrier was observed. Therefore, overall 4.8% (3/62) of the tested individuals belonging to the Northern Finnish 1997–2011 study cohort turned out to be carriers of the *PALB2* c.1592delT allele.

**Conclusions:**

Given the potential benefits versus harms of this testing, the result of our study suggest that *PALB2* c.1592delT should be a routine part of the genetic counseling protocol for Finnish high-risk breast cancer cases tested negative for mutations in *BRCA1/BRCA2*.

## Background

It has been estimated that about 5-10% of all breast cancer cases are caused by dominantly inherited mutations [1]. *BRCA1* and *BRCA2* are clinically the most important genes associated with breast cancer susceptibility [2,3]. Depending of the studied population, heterozygous mutations in these two high-penetrance cancer genes are responsible for 20-30% of familial breast cancer cases [2,3]. A meta-analysis of 22 studies by Antoniou and colleagues [4] found the cumulative risk of breast cancer by age 70 to be 65% in *BRCA1* and 45% in *BRCA2* mutation carriers. At cellular level, BRCA1 and BRCA2 have many important functions integral for a successful response to DNA damage [5]. Many other genes have also been implicated in breast cancer predisposition due to their essential role in the same pathway required to maintain genomic integrity. Among them, *PALB2* (partner and localizer of BRCA2) has emerged as a breast cancer susceptibility gene in several populations world-wide [6]. PALB2-BRCA2 interaction is essential for the DNA double-strand break repair functions of BRCA2 [7] and PALB2 also functionally connects the BRCA1 and BRCA2 proteins [8]. Furthermore, PALB2 is an important regulator of homologous recombination [9]. The biological functions of BRCA proteins have led to clinical trials testing targeted therapies, particularly those utilizing PARP (poly-ADP-ribose polymerase) inhibitors [10]. PARP is an important regulator of the DNA base-excision –repair pathway [11]. Analogous to BRCA1/2-deficiency, PALB2-deficient cells are sensitive to PARP inhibitors [9].

Individuals who carry germline mutations in the *PALB2* gene are at increased risk of breast cancer [12]. *PALB2* mutations have also been linked to pancreatic cancer in families with concomitant breast cancer [13]. The Finnish founder mutation c.1592delT in exon 4 causes a frameshift on Leu531 and premature truncation of the PALB2 protein [14]. This mutation is present at a significantly elevated frequency in familial breast cancer cases compared with ancestry-matched population controls. Also the studied unselected breast cancer cohort showed a roughly 4-fold enrichment of the mutation compared with controls [14]. The c.1592delT mutation is associated with a 6-fold increased risk of breast cancer; equivalent to a ~40% risk for developing breast cancer by the age of 70 years. This is comparable to that for carriers of mutations in *BRCA2*[15]. The average age for the onset of breast cancer in *PALB2* c.1592delT carriers is 53.1 years, which is somewhat higher than in Finnish *BRCA1/BRCA2* mutation carriers (45.2/47.4 years), but considerably lower than in sporadic patients (58.0 years) [14]. Furthermore, the breast tumors of *PALB2* carriers manifest a phenotype associated with aggressive disease, and mutation carriers also display reduced survival [16].

The purpose of genetic counseling is to allow individuals an opportunity to learn how heredity contributes to their personal risk of developing cancer. The key to the identification of individuals who will benefit from genetic testing for hereditary cancer susceptibility genes lies in the risk assessment process. It is well established that patients should be offered a molecular analysis of the *BRCA1* and *BRCA2* genes when (1) the family history is suspicious for a cancer predisposition syndrome; (2) the results can be adequately interpreted; and (3) the results will influence medical management [17]. The aim of this study was to evaluate whether *PALB2* c.1592delT mutation testing should be part of routine clinical counseling in families fulfilling the criteria for *BRCA1/BRCA2* testing, but found negative for mutations in these genes. The sensitivity of our current *BRCA1*/*BRCA2* mutation analysis protocol was also defined for genetic counseling purposes. Here we have screened affected index persons of Northern Finnish high-risk hereditary breast and/or ovarian cancer families for presence of the *PALB2* c.1592delT mutation.

## Methods

### Patient material

Subjects were selected among the individuals who had been in contact with the Oulu University Hospital, Department of Clinical Genetics between years 1997 and 2011. Altogether 223 individuals contacted the outpatient clinic because they or their first-degree relative had been diagnosed with breast and/or ovarian cancer. Inclusion for the current study was done according to the following criteria of high-risk hereditary breast cancer (the Lund criteria): (1) the individual or her first-degree relative (only female family members were included when defining first-degree relatives) had breast and/or ovarian cancer at an age younger than 30 years; or (2) two first-degree relatives in the family had breast and/or ovarian cancer and at least one of the cancers had been diagnosed at an age younger than 40 years; or (3) three first-degree relatives in the family had breast and/or ovarian cancer and at least one of the cancers had been diagnosed at an age younger than 50 years; or (4) four or more relatives had breast and/or ovarian cancer; or (5) the same individual had both breast and ovarian cancer. Individuals with bilateral breast cancer were considered having two separate cancers. *BRCA1* or *BRCA2* positive mutation status was an exclusion criteria: altogether 16 individuals were excluded because they or their close relative was tested positive for a *BRCA* mutation.

**Figure 1:**
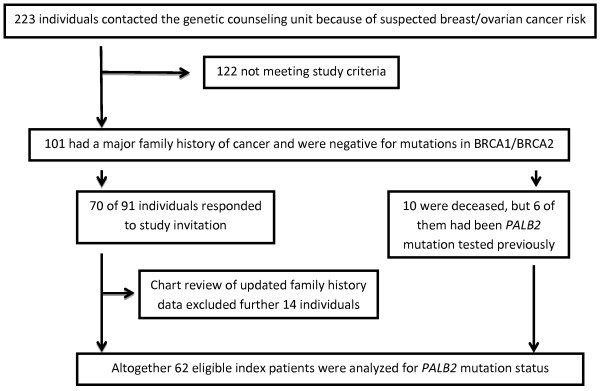
**Flow diagram summarizing the strategy for identifying *PALB2 *c.1592delT mutation carriers in a Northern Finnish 1997–2011 study cohort consisting of high-risk breast cancer family index patients that had tested negative for mutations in *BRCA1*/*BRCA2*.**

Of the initial 223 individuals, 101 met the inclusion criteria based on their pedigree data. Of the eligible persons, 10 were now deceased but 6 of them had participated in one of our previous studies on *PALB2*[14]. The remaining 91 were alive at the time of ascertainment and were invited by a letter to participate in our study. Seventy individuals responded (70/91 = 77%) and provided us with information on their family history of cancer. Chart review led to the further exclusion of 14 individuals as they did not meet the selection criteria. Altogether the current Northern Finnish 1997–2011 study cohort that was tested for *PALB2* c.1592delT carrier status consisted of 62 (56 alive and 6 deceased) affected female individuals (see Figure 1). Informed consent had been obtained from all participants. After completed laboratory analysis, the patients still alive were informed about their mutation status, if they had requested this information. Appropriate genetic counseling was arranged if desired. The study protocol was approved by the Ethical Committee of the Oulu University Hospital (12/2000 and 12/2011).

### Mutation detection

Fifty-six well-characterized high-risk hereditary breast and/or ovarian cancer *BRCA1*/*BRCA2* mutation-negative Northern Finnish individuals were screened for the *PALB2* c.1592delT germline mutation. The mutation screening was performed on peripheral blood DNA by using high resolution melting (HRM) analysis and direct sequencing. Samples with deviations in their melt curves in HRM were reamplified and sequenced with capillary sequencing using ABI3130x1 Genetic Analyzer (Applied Biosystems, Foster City, CA, USA). For ABI the Big dye terminator kit v1.1 (Applied Biosystems, Foster City, CA, USA) was used. Chromatograms were interpreted using CodonCode Aligner v.3.5.4 (Codon Code Corporation, Dedham, MA, USA) and MEGA4 software [18]. Oligonucleotides for HRM and sequencing were designed using Primer3 software [19]. Primer sequences, detailed HRM and PCR reaction conditions are available upon request. *PALB2* mutation detection for the 6 eligible but now deceased 1997–2011 study cohort persons was carried out as mentioned in our previous study [14].

Information on tumor histology, grade, size, nodal status and distant metastasis were collected from pathology reports. Estrogen receptor and progesterone receptor status were confirmed by a breast cancer pathologist. Evaluation of the results and the staining methods used in routine diagnostics has been described by Eerola et al. [20].

## Results

Of the 56 alive index patients, two carried the *PALB2* c.1592delT mutation (2/56 = 3.6%). In addition, among the 6 previously tested but now deceased persons eligible for the current study, one more mutation carrier was observed. Therefore, overall 4.8% (3/62) of the individuals belonging to the Northern Finnish 1997–2011 study cohort turned out to be carriers of the *PALB2* c.1592delT allele. The observed frequency is slightly higher to that reported in the previous study, where the c.1592delT mutation was found in 2.6% of familial breast cancer cases with a strong family history of the disease [14]. The difference is probably due to the use of even more stringent inclusion criteria for the patients participating in the current study.

**Figure 2:**
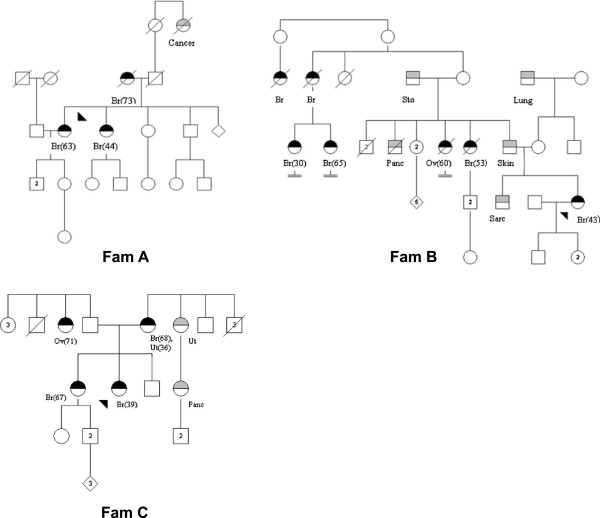
**Pedigrees of the three families (A-C) positive for the studied *PALB2 *mutation.** Patients with breast or ovarian cancer are marked with half-filled black circles, and persons with other malignancy types with circles (females) or squares (males) half-filled with gray. Br, breast cancer; Cancer, cancer of unknown type for which no pathology reports were available; Lung, lung cancer; Ov, ovarian cancer; Panc, pancreatic cancer; Sarc, sarcoma; Skin, skin cancer; Sto, stomach cancer; Ut, uterus cancer. (x) age at diagnosis, if available. Arrow marks index.

The cancer types of the three observed *PALB2* mutation positive individuals fitted well to that described previously [16]: all three cases had ductal tumor histology and exhibited positive estrogen and progesterone receptor status (Table 1). Besides breast cancer, two of the families also displayed several other malignancy types, including ovarian, pancreatic and uterus cancer (see Figure 2 for family pedigrees).

**Table 1:** **Tumor characteristics of identified *PALB2 *c.1592delT mutation carriers**

**Family id**	**Cancer (age)**	**Histology**	**TNM**	**ER/PR status**	**Grade**	**Her2**	**p53**	**Ki67**	**Other cancer cases in the family**
Fam A	Br(63)	Ductal	T2N0M0	ER pos/PR pos	2	Neg	Neg	2	Br(44), Br(73), Cancer
Fam B	Br(43)	Ductal	TisN0M0	ER pos/PR pos	1	Neg	NA	0	Br(30), Br(53), Br(65), Br, Br, Lung, Ov(60), Panc, Sarc, Skin, Sto
Fam C	Br(39)	Ductal	T2N0M0	ER pos/PR pos	3	NA	Neg	2	Br(67), Br(68), Ov(71), Panc, Ut(36), Ut

*ER*, Estrogen receptor; *PR*, Progesterone receptor; *Her2*, Human epidermal growth factor receptor 2; *p53*, Tumor protein 53; *Ki67*, Antigen Ki67; *Br*, Breast; *Cancer*, Cancer of unknown type for which no pathology reports were available; *Ov*, Ovarian; *Panc*, Pancreatic; *Sarc*, Sarcoma; *Sto*, Stomach; *Ut*, Uterus; *NA*, Not available.

The currently identified mutation positive individuals still being alive were then invited for genetic counseling, where they were also offered the possibility of more careful cancer surveillance. An opportunity for genetic counseling was offered also to their family members.

## Discussion

The identification of breast cancer susceptibility genes, particularly *BRCA1* and *BRCA2*, has revolutionized the management of women with a family history of the disease [2]. Individuals undergoing cancer genetic counseling must understand the benefits, limitations and risks of genetic testing. If a deleterious mutation is identified, it allows for predictive testing in at-risk family members [17]. However, at this moment, if no mutations in these two susceptibility genes are identified, usually no more genetic testing for additional rare cancer-predisposing syndromes is offered in Finland, unless indicated by specific findings and/or family history. Consequently, the members of these *BRCA1/2*-negative high-risk families lack any specific information about their individual risks. However, as the *PALB2* c.1592delT founder mutation, with a frequency of roughly 1% also in Finnish breast cancer cases unselected for their family history of disease, increases the risk of breast cancer in a way comparable to that of *BRCA2*[21], targeted clinical testing for c.1592delT seems appropriate. It has also been suggested that a more careful presymptomatic screening, as well as more aggressive adjuvant treatment, should be considered for *PALB2* c.1592delT carriers [16]. Of the currently studied 62 female index individuals who had tested negative for germline mutations in *BRCA1*/*BRCA2*, 4.8% were *PALB2* mutation carriers. Therefore, approximately the same proportion of the high risk patients, together with their family members, could be offered more specific genetic counseling and predictive testing if *PALB2* c.1592delT testing would be a routine part of the national counseling protocol.

Testing for a single and also relatively well-characterized founder mutation has obvious benefits. The molecular analysis for *PALB2* c.1592delT is very cost-efficient. Women who test positive for a known gene mutation can make informed decisions about their cancer surveillance, and women with a negative result can avoid unnecessary disease monitoring. Furthermore, targeted therapies may be available for mutation carriers in the future. A deeper understanding of the function of PALB2 may aid the design of therapeutic strategies for cancer treatment, such as the use of PARP inhibitors. The identification of yet additional families with this as well as other *PALB2* mutations will lead to better estimates of the associated cancer risks and to better understanding of the optimal surveillance and prophylactic regimens for mutation carriers.

## Conclusions

Heterozygosity for *PALB2* c.1592delT has a strong effect on breast cancer risk. The mutation is also common enough to make it worthwhile to set up a simple diagnostic test that should be offered as part of genetic counseling to all Finnish high-risk breast cancer cases negative for mutations in *BRCA1* and *BRCA2*. The same recommendation would most likely apply also to other high-risk *PALB2* founder mutations present in other populations world-wide.

## Competing interests

The authors declared that they have no competing interest.

## Authors’ contributions

MH, KP, JSM and RW conceived and designed the study. MH and KP carried out the data analysis. MH performed the genetic counseling and drafted the manuscript, and all authors contributed to the subsequent versions of the manuscript. All authors read and approved the final version of the manuscript.

## Pre-publication history

The pre-publication history for this paper can be accessed here:

http://www.biomedcentral.com/1471-2350/14/82/prepub
